# Fetal Right Ventricular Diverticulum Detected by Prenatal Ultrasound Screening

**DOI:** 10.1155/2016/6382920

**Published:** 2016-10-12

**Authors:** Daisuke Katsura, Kaori Hayashi, Shunichiro Tsuji, Tetsuo Ono, Akiko Ishiko, Kentaro Takahashi, Takashi Murakami

**Affiliations:** Department of Obstetrics and Gynecology, Shiga University of Medical Science Hospital, Seta Tsukinowa-cho, Otsu, Shiga 520-2192, Japan

## Abstract

Prenatal ultrasound screening has allowed for the detection of in utero cardiac abnormalities. Specifically, distinction is possible between ventricular diverticula and aneurysms, which is important because each condition has a different clinical outcome. We report the case of a 35-year-old, gravida 1, para 1 woman, with no significant past medical history, who underwent routine prenatal ultrasound screening at 32 weeks' gestation. A four-chamber ultrasound of the fetal heart combined with M-mode echocardiography showed abnormal dilatation of the right ventricular chamber measuring 2.2 cm × 1.0 cm but with normal contractility. Delivery was performed at full term by cesarean section, and a right ventricular diverticulum was confirmed by postnatal cardiac computed tomography. The baby developed normally with no cardiac sequelae during followup. This case demonstrates the importance of making a correct diagnosis of ventricular diverticula by prenatal ultrasound when abnormal dilatation of the fetal ventricle is identified during routine screening. Because evaluating the wall contractility by M-mode ultrasound leads to evaluating whether it has the myocardium, we conclude that M-mode echocardiography is effective for the purpose of prenatal cardiac diagnosis and can distinguish between ventricular aneurysms and functioning ventricular diverticula.

## 1. Introduction

Ventricular diverticula are rare, with an estimated incidence of 0.013% [1]. Embryonic developmental disturbance, myocardial infection, and myocardial infarction are thought to be the causative factors [1, 2], and the prognosis is good in isolated cases. However, complications include rupture, arrhythmia, thrombus formation, heart failure, and infective endocarditis; therefore, they typically require monitoring [1, 3].

Ventricular diverticula are classified as muscular or fibrous [4, 5]. The former is composed of three cardiac layers, including the internal membrane, myocardium, and outside membrane. Crucially, these have normal systolic contraction and a narrow connection to the ventricle and are also usually located at the ventricular apex. Muscular diverticula may be associated with other intracardiac anomalies and midline thoracoabdominal defects. By contrast, fibrous diverticula are composed of fibrous tissue with residual myocardial fibers. Consequently, these may be associated with akinetic or dyskinetic contraction, may have a broad connection to the ventricle, and are usually connected to subvalvular areas as an isolated lesion. Fibrous diverticula can, therefore, be considered synonymous to ventricular aneurysms.

The 10-year survival rate of ventricular diverticula is approximately 80%, whereas the 4-year survival rate of ventricular aneurysms is approximately 30% [6]. When an abnormal heart chamber is noted to be continuous with the ventricle by fetal ultrasound screening, the need to distinguish between a ventricular diverticulum and aneurysm is important. However, as observed by Williams et al. [7], this can be extremely difficult, especially when relying on brightness-mode (B-mode) ultrasound used for fetal screening. Although there are several case reports of ventricular diverticula, these relied on B-mode ultrasound only, and it is unclear whether the cases were adequately distinguished from ventricular aneurysm [8–10]. In this report, we present a case of a right ventricular diverticulum diagnosed prenatally by motion-mode (M-mode) ultrasonography.

## 2. Case Presentation

**Figure 1:**
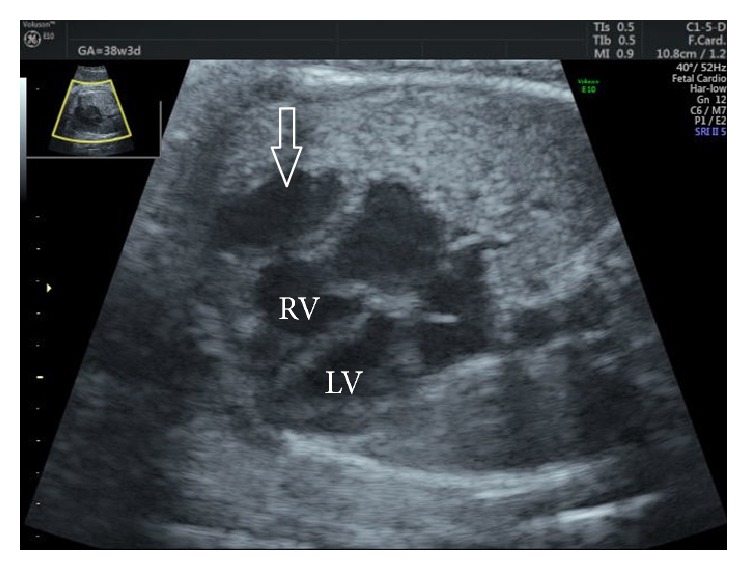
Fetal ultrasound: four-chamber view showing the diverticulum arising from the right ventricle. LV, left ventricle; RV, right ventricle.

**Figure 2:**
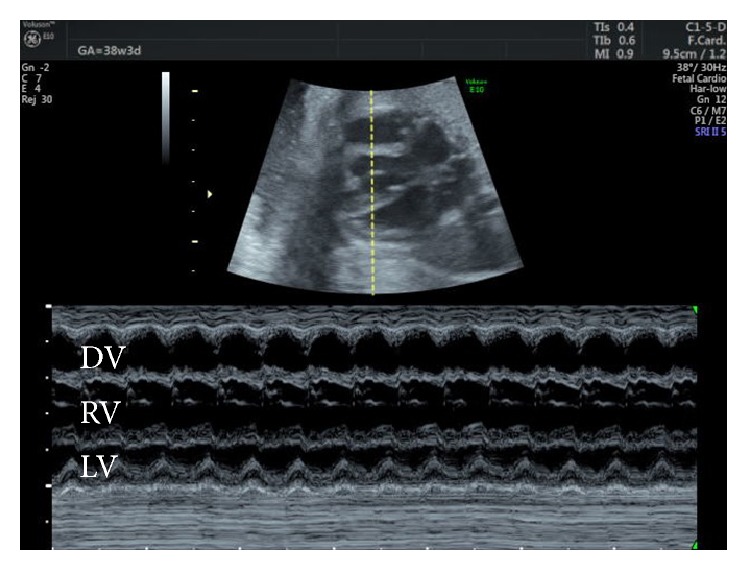
Fetal M-mode ultrasound: four-chamber view showing the diverticulum, right ventricle, and left ventricle. DV, diverticulum; LV, left ventricle; RV, right ventricle.

This case involves a 35-year-old, gravida 1, para 1 mother with unremarkable medical and family histories. At routine pregnancy checkups, an abnormal fetal heart chamber was identified in the 32nd week of pregnancy and she was referred to our hospital in the 35th week of pregnancy. At that time, she was noted to have an abnormal heart chamber (2.2 cm × 1.0 cm) that was continuous to the front and outside of the right ventricle on four-chamber fetal ultrasound screening (Figure 1). The presumptive diagnosis at this point was either a ventricular diverticulum or a ventricular aneurysm. The connection to the ventricle was broad; however, color Doppler ultrasound detected blood flow in and out of the abnormal heart chamber, and there was normal synchronous contraction of its walls with those of the ventricle by M-mode assessment (Figure 2). The heart function was otherwise normal. No other cardiac or extracardiac anomalies were detected, and fetal growth was within normal limits for the gestational age. Therefore, we diagnosed a right ventricular diverticulum.

**Figure 3:**
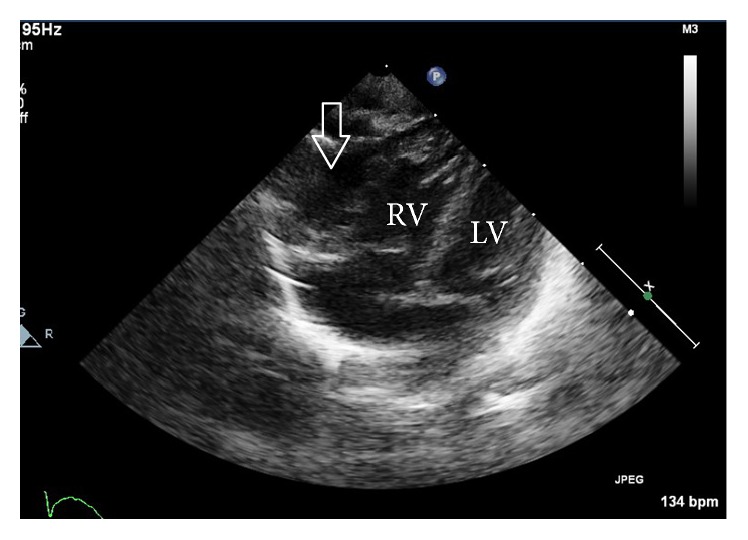
Postnatal ultrasound: four-chamber view showing the diverticulum arising from the right ventricle. LV, left ventricle; RV, right ventricle. Arrow indicates the diverticulum.

**Figure 4:**
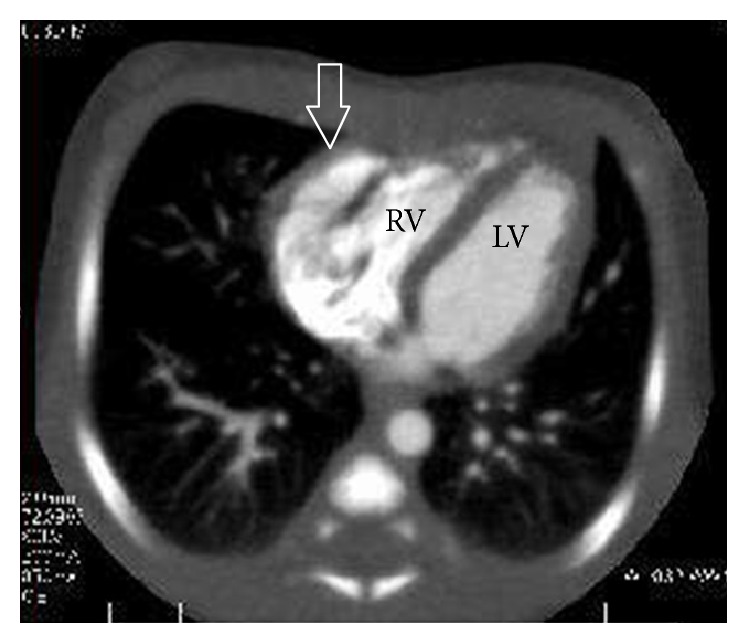
Postnatal computed tomography scan showing myocardium in the diverticulum arising from the right ventricle. LV, left ventricle; RV, right ventricle. Arrow indicates the diverticulum.

The subsequent course of the pregnancy was uneventful until term, when an elective cesarean section was performed in the 38th week because of a past cesarean section. A male infant was born weighing 2982 g, with an Apgar score of 8 at 1 min and 9 at 5 min and an umbilical arterial blood pH of 7.275. After the delivery, he was diagnosed definitively with a right ventricular diverticulum by the presence of myocardium in the abnormal heart chamber on computed tomography, with continued normal contraction (Figures 3 and 4). His prognosis was considered good, and he was discharged without heart symptoms and without turbulent flow or thrombus in the diverticulum. At the time of this writing, he is 6 months of age and remains asymptomatic without medication. He is attending regular followup at our hospital.

## 3. Discussion

Generally, B-mode ultrasound is used for fetal screening, producing a grayscale picture that expresses the difference in acoustic impedance in black and white to indicate differences in “brightness.” This offers excellent visibility and is appropriate for observing the four-chamber view. However, the distinction between ventricular diverticula and aneurysms is difficult when using only B-mode ultrasound.

When evaluating the wall thickness of the abnormal heart chamber by B-mode ultrasound, Peters et al. [11] proposed that diverticula could be diagnosed if the wall was thick and aneurysm if the wall was thin. When an abnormal heart chamber is identified in the first trimester or when it remains very small in the second trimester, estimating the wall thickness can be extremely difficult. In other research, McAuliffe et al. [12] and Koshiishi et al. [10] distinguished aneurysms from diverticula by the size of the orifice connecting the ventricles. In addition to this criterion, Cavallé-Garrido et al. [13] and Del Río et al. [14] included the evaluation of abnormal heart chamber contractibility. Because the important point in the distinction between ventricular diverticulum and aneurysm is whether an abnormal heart chamber has the myocardium, evaluating the wall contractility leads to evaluating whether it has the myocardium. In addition, the distinction by the orifice size alone would probably be inadequate as observed in this case. Therefore, we believe that this is more appropriate. Equally, although other researchers distinguished aneurysms and diverticula by evaluating contractibility [5, 6, 15–17], they concluded that it was difficult to evaluate it by only the B-mode method.

Ultrasonography also includes the facility for M-mode imaging, in which the picture can display movement and change over time as the strength and weakness of the ultrasound beam's brightness on the same line continuously. In adults or newborns, echocardiography is frequently used to observe movement of the heart muscle and its valve, and it can determine whether there is myocardial contraction. Thus, it is possible that an abnormal heart chamber may be diagnosed as a ventricular diverticulum if normal contraction is present or a ventricular aneurysm if akinetic or dyskinetic contraction is present when evaluating the contractility of the abnormal heart chamber wall. In our case, the abnormal heart chamber was diagnosed prenatally as a ventricular diverticulum by confirming that the wall had normal contraction on M-mode ultrasound, which was later confirmed by postnatal computed tomography. Demir et al. [18] and Olorón et al. [19] also evaluated wall contractility using M-mode ultrasound, supporting the use of this method for distinguishing between these disorders. In addition, because muscular diverticulum has normal contractions, color Doppler ultrasound can detect blood flow from the diverticulum to the heart ventricle for diastole of the heart and from the heart to the diverticulum for systole. This method as well as M-mode ultrasound may be useful for evaluating wall contractility. As aforementioned, for distinction between ventricular diverticulum and aneurysm, we recommend evaluation of the wall thickness by B-mode ultrasound and the wall contractility by M-mode ultrasound and color Doppler. However, because Ohlow et al. [20] reported that 8% of ventricular aneurysms had normal contractility and that 10% of ventricular diverticula were akinetic, clinicians must make an overall judgment based on other criteria. Indeed, in this study, we diagnosed the ventricular diverticulum by reliance on both the thickness and contractility of the abnormal heart chamber, as previously supported.

Recently, speckle tracking has been reported to be of value for assessing movement of heart walls [21]. This technique calculates the local velocity and strain in tissue by following the movement of a punctate structure (speckle pattern) in each frame during the B-mode method [22]. Only information on the ultrasonic line in M-mode is acquired, allowing evaluation of the entire motion if speckle tracking is used. Moreover, speckle tracking may be effective for the evaluation of ventricular diverticula with fibrous parts or small abnormal heart chambers that are difficult to evaluate by M-mode ultrasound. Although it is expected that the precision of fetal diagnoses will improve with technological advances, current M-mode ultrasound is sufficiently effective for prenatal diagnosis of ventricular diverticula in the meantime.
